# An Interactive Mock Paging Curriculum to Prepare New Internal Medicine Interns for Inpatient Wards

**DOI:** 10.15766/mep_2374-8265.11082

**Published:** 2021-01-13

**Authors:** Rima Patel, Laura K. Snydman

**Affiliations:** 1 Resident Physician, Department of Medicine, Tufts Medical Center; 2 Associate Professor, Department of Medicine, Tufts Medical Center

**Keywords:** Internal Medicine, Curriculum, Small-Group Learning, Curriculum Development, Hospital Medicine, Case-Based Learning

## Abstract

**Introduction:**

The July effect refers to an increase in adverse outcomes during periods of physician trainee turnover in teaching hospitals. We created an interactive resident-led curriculum to train new internal medicine interns for routine encounters on inpatient wards by role-playing through mock paging scenarios and focusing on practical information relevant to intern year.

**Methods:**

A formal assessment of the academic year 2018 intern boot camp curriculum revealed that interns preferred sessions that involved active learning strategies and covered common issues. In the first week of academic year 2019, interns participated in two 1-hour small-group sessions involving mock paging scenarios. Interns were divided into small groups with one facilitator who was a senior medicine resident. Within these groups, facilitators acted as the nurse and provided pages. Interns took turns answering these mock pages based on a sign-out of patients. The facilitator emphasized desired learner actions and teaching points using a provided guide.

**Results:**

Twenty interns participated in the curriculum. Interns rated the curriculum highly and felt that the sessions improved their knowledge, comfort, and skills in managing routine inpatient encounters. On a 2-week follow-up knowledge test to determine if they retained the information from the curriculum, interns scored an average of 85% (response rate: 60%, *N* = 12), indicating that they could apply the knowledge/skills learned to new scenarios.

**Discussion:**

This curriculum prepares medicine interns to manage common inpatient issues at the beginning of their residency. After completing the curriculum, interns reported increased confidence in handling these issues.

## Educational Objectives

By the end of this curriculum, learners will be able to:

1. Demonstrate knowledge and skills in responding to common pages on inpatient medicine wards.
2. Identify key tasks, including relevant medication and laboratory orders, involved in managing acute scenarios.
3. Describe when an acute medicine issue requires immediate attention and consultation with a senior resident.
4. Demonstrate increased self-perceived comfort with managing acute issues on medicine wards.

## Introduction

It is widely known that there is heightened concern about patient care during academic year turnover, when medical students across the country transition to interns. There have been reports of an increase in undesirable events at the beginning of the academic year compared to the rest of the year^1^ and increased mortality for patients during this turnover period.^2^ A systematic review demonstrated increased mortality and decreased efficiency during this time of changeover.^3^ This well-known July effect refers to the increase in adverse outcomes during periods of physician trainee turnover in teaching hospitals nationwide.^4^ There is a rigorous need to improve preparation of future residents in order to reduce these adverse outcomes.^5,6^

Fourth-year medical students often undergo boot camp curricula to help them prepare for internship. These curricula review various topics such as surgical techniques, radiology skills, calling consultants, electrocardiogram interpretation, and management of acute scenarios.^7–11^ Such curricula have been shown to help medical students feel more prepared for residency and to increase their confidence.^12,13^ However, despite these boot camps, objective assessments at the beginning of internship have demonstrated gaps in new interns’ knowledge and skills, largely due to variable undergraduate medical education experiences.^5,14^ In addition, in the months preceding residency, fourth-year medical students are often travelling, conducting research projects, participating in global health electives, and/or partaking in other activities that place them away from clinical experience. In a survey of fourth-year medical students’ perspectives on an ideal curriculum for their last year, students valued activities related to securing their chosen residency, opportunities to take electives, and vacation time.^15^ Thus, the fourth year of medical school may not be the most ideal time to teach residency preparedness skills. In contrast, interns’ priorities are focused on succeeding in residency as they face greater responsibilities as new physicians. In addition, when interns are taught residency preparedness skills, they can immediately and independently apply those skills, which is not the case for fourth-year medical students.

Based on our review of the literature, there currently are curricula to train interns on surgical issues, effective patient hand-offs, end-of-life decision-making, and the ACGME General Competencies, among others.^16–19^ One curriculum concentrates on improving interns’ management of common internal medicine skills, such as efficiency and team communication skills, but it does not explicitly focus on practical information to help interns manage acute paging scenarios.^20^ Its sessions also span more than 3 months, yet interns may require some of these skills earlier in the year. Other workshops use high-fidelity mannequin simulation to train residents on acute scenarios in the critical care setting.^21,22^ However, high-fidelity mannequin simulation is not readily available at every institution and may be difficult for some programs to obtain.

Our goal was to design a curriculum specifically for internal medicine interns, focusing on building their skill set for handling acute scenarios on inpatient wards and communicating with nurses at the beginning of their training. We sought to achieve this by creating an interactive curriculum training new interns to appropriately manage routine nurse pages by role-playing through mock pages and incorporating practical information geared towards the start of intern year.

## Methods

This project was reviewed by the Institutional Review Board at Tufts Medical Center and deemed to be exempt (Protocol no. 13527).

### Curriculum Development

Our institution's internal medicine program provides new interns with a lecture-based curriculum in their first month of training. This curriculum consists of weekly 1-hour lectures on various topics such as antibiotics, dyspnea, gastrointestinal bleeding, chest pain, shock, end-stage renal disease, cardiac devices, and sepsis. We conducted a survey-based needs assessment of the academic year 2018 interns at our institution on the usefulness of this curriculum. The response rate was 56% (14 out of 25). Interns rated how strongly they agreed with each aspect of the curriculum using a 5-point Likert scale (1 = *strongly agree,* 2 = *agree,* 3 = *somewhat agree,* 4 = *disagree,* 5 = *strongly disagree*). Interns found the existing curriculum useful (median = 2) but preferred a more memorable format using active learning strategies (median = 3). When asked which teaching styles they found most effective, most interns (56%) preferred small-group problem-based learning. The sessions they found most useful were those involving interaction and commonly encountered ward issues and themes such as chest pain or shortness of breath rather than specific uncommon topics such as cardiac devices or transplant issues.

Based on our needs assessment, we created an interactive curriculum addressing common ward issues interns encounter. To establish the content of the curriculum, we surveyed the academic year 2018 intern class and had a response rate of 80% (16 out of 20). Interns were asked to rate how frequently they were paged about common ward issues and how comfortable they felt managing each of these pages at the beginning of their intern year. Interns reported being paged from most to least frequently about pain, blood sugar, chest pain, fever, shortness of breath, and hypotension (Table). Some interns also listed difficulty sleeping, hypertension, and tachycardia as frequent pages. Interns were least comfortable with pages they received less frequently (e.g., hypotension, shortness of breath, and chest pain).

**Table.**
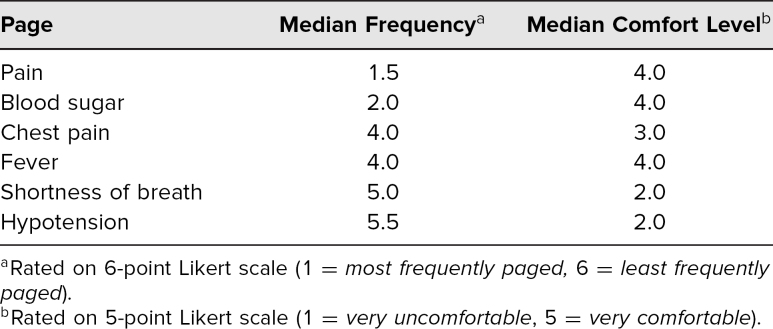
Needs Assessment of Most Frequent Pages That Interns Receive

Based on the survey results, we developed 12 mock paging scenarios involving these high-yield common ward issues. After the scenarios were created, feedback was obtained from attending physicians and senior internal medicine residents. The scenarios were piloted with a group of fourth-year medical students in their internal medicine subinternship. They provided positive feedback on the curriculum and described the difficulty level as appropriate.

The curriculum consisted of intern and facilitator guides for each session. The intern guides (Appendices A [Day 1] and B [Day 2]) outlined session goals, session structure and time line, and interns’ roles in the scenario, as well as including a sign-out of mock patients. The facilitator guides (Appendices C [Day 1] and D [Day 2]) outlined session goals, session structure and time line, and the facilitator role. The facilitator guides also included the mock pages, notes for the nurse (facilitator), desired learner actions, and teaching points relevant to each case. Appendix E consisted of an EKG used as supplemental material for a mock paging scenario in the first session.

### Participants

Twenty internal medicine interns at our institution participated in the curriculum as part of their internal medicine residency training. The sessions were conducted in the first week of their intern year at the start of academic year 2019.

The session facilitators were internal medicine chief residents and senior residents who volunteered to proctor the sessions. All facilitators were provided with instructions on the general structure of the session, their role as facilitator, and facilitator guides (Appendices C and D) prior to the session to familiarize them with the curriculum content.

### Session Structure

The curriculum consisted of two 1-hour sessions on 2 consecutive days during the first week of intern year. The sessions were held during dedicated time interns had for didactics. Six mock paging scenarios were discussed during each session. Prior to the session, interns were divided into small groups of four to five interns led by a facilitator who was either a senior or chief internal medicine resident. All interns received a copy of the intern guides (Appendices A [Day 1] and B [Day 2]), and all facilitators received a copy of the facilitator guides (Appendices C [Day 1] and D [Day 2]) and associated materials (Appendix E).

During the first 5 minutes of the session, the session structure and time line were discussed with the group. Interns were also provided with key points to keep in mind when answering the mock pages, as described in the intern guides (Appendices A and B). Effective communication techniques with nurses were also discussed, using the examples in the beginning of the intern guide (under “Role as Intern” in Appendices A and B).

Each group then went through each of the mock paging scenarios. Within each small group, the facilitator acted as the nurse and gave the interns a mock page by reading it from the facilitator guide. Interns took turns acting as a cross-covering intern and answered the pages based on the provided sign-out of patients. Facilitators were instructed to provide only information the interns asked about. They prompted the interns to complete desired actions as necessary and encouraged them to practice effective communication. Following each mock page, the facilitators discussed relevant teaching points included in their guide and answered any questions. For each case, they also discussed what type of documentation would be appropriate. The facilitator and other interns provided feedback to the intern answering the page. In order for the session to be completed in the allotted time of 1 hour, suggested times were provided for each scenario, but each group had the flexibility of moving at its own pace. The last 5 minutes of the session were used to provide general feedback and answer any remaining questions. The session structure for the second day was the same as for the first day.

### Session Evaluation

Immediately following the curriculum, interns completed a session evaluation form (Appendix F). This survey asked interns about the curriculum's usefulness, difficulty level, and applicability to inpatient medicine wards. Using a retrospective pre-post test design, interns self-reported their comfort and knowledge in managing common ward issues before and after the sessions using a 5-point Likert scale.

Interns received a knowledge test (Appendix G) electronically by email 2 weeks after the curriculum to assess if they had retained the material discussed. A focus group of attending physicians and internal medicine residents reviewed the knowledge test and adjusted the difficulty level and number of questions to better cater to interns at the beginning of the academic year. Criteria for correct and incorrect answers were predetermined (Appendix H) based on feedback from this focus group. The final knowledge test consisted of nine novel mock paging scenarios similar to those in the curriculum and assessed interns’ knowledge of key concepts reviewed during the sessions.

## Results

**Figure 1.**
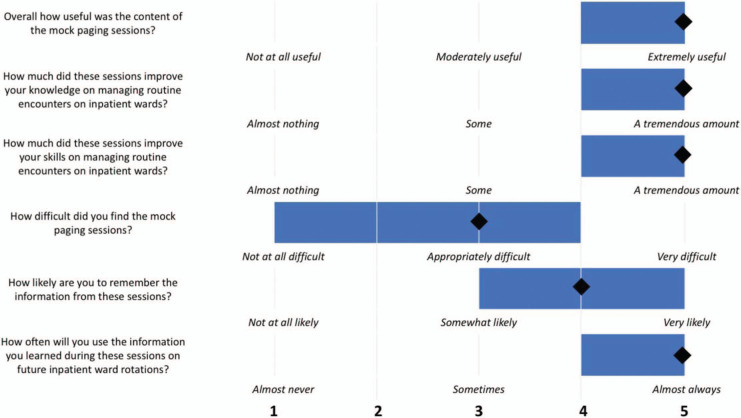
Results of the session evaluation. Based on 5-point Likert scales as delineated above, interns (*n* = 20) responded to questions about the curriculum following completion. Blue bars denote ranges of scores, and diamonds represent median responses.

Twenty internal medicine interns at our institution participated in the academic year 2019 curriculum. Immediately following the curriculum, interns completed the session evaluation (Appendix F) with a 100% response rate. Interns rated the overall quality of the session as excellent (median = 5, where 1 = *poor,* 2 = *fair,* 3 = *good,* 4 = *very good,* and 5 = *excellent*). In the first part of the survey, interns responded to various questions about the curriculum using a 5-point Likert scale. Figure 1 depicts the median responses to each of these questions. Overall, interns found the content of the mock paging sessions to be very useful. They felt that the sessions improved their knowledge and skills in managing routine inpatient nurse pages. The difficulty level of the mock paging scenarios was considered appropriate. Interns also felt that they would remember the information in the future and be able to apply it to inpatient wards.

**Figure 2.**
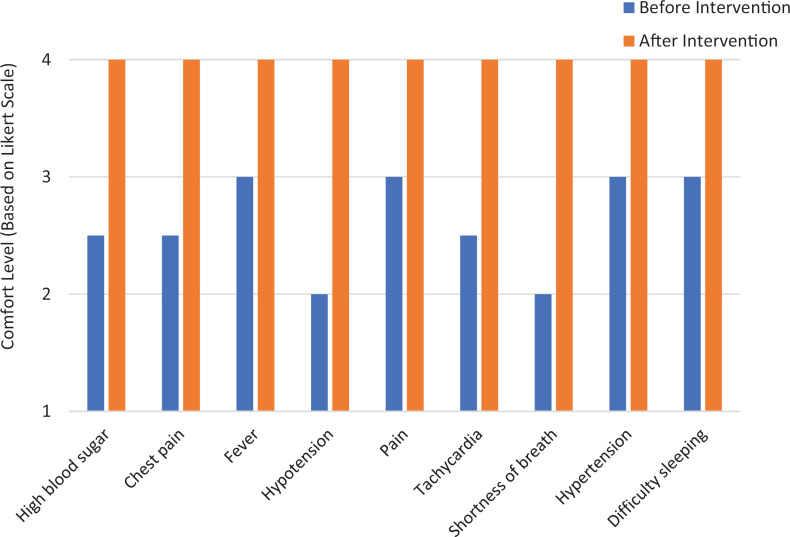
Interns’ median comfort level with common inpatient ward issues before and after the curriculum. After completion, interns (*n* = 20) retrospectively reported their comfort level with common ward issues before the intervention as well as after using a 5-point Likert scale (1 = *very uncomfortable,* 5 = *very comfortable*).

The second part of the survey asked interns to compare how comfortable they were managing common ward issues (high blood sugar, chest pain, fever, hypotension, pain, tachycardia, shortness of breath, hypertension, difficulty sleeping) before and after the curriculum. As shown in Figure 2, interns felt more comfortable handling these issues after the curriculum. Based on a Wilcoxon signed rank test, the median difference in interns’ comfort levels with these issues before and after was statistically significant (*p* = .0003).

When interns were asked what they enjoyed about these sessions, most of their comments focused on the interactive nature of the session and the practicality of the information discussed. Examples of their statements include the following:

• “Found this extremely useful. It helped to go over the specific actions we should take in terms of what orders to put in and what to ask the nurse for.”
• “Small groups with great teachers; able to get specific questions answered.”
• “Practical, real and interactive. I will likely remember what I got from it.”
• “Small groups worked well. It was great that we each got a chance to practice. Also great to have a senior walk us through things in detail.”
• “Real life applicable situations that we will encounter—thinking through solutions in safe, controlled environment.”
• “These are real life situations that could happen and made me feel more comfortable before my night float.”

In regard to which aspects of the curriculum they would change, several interns suggested having the sessions as early as possible in intern year. They did not report that any of the discussed topics should be removed. Two interns suggested including altered mental status as an additional issue to cover.

A knowledge test (Appendix G) was administered at 2 weeks to determine if interns had retained the material discussed. This knowledge test consisted of nine novel mock paging scenarios with a total of 12 questions. For each scenario, there were open-ended questions about next steps in diagnosis and/or treatment, including medication orders, laboratory testing, and imaging. Twelve interns responded to this knowledge test, for a response rate of 60%. All knowledge tests were graded by the first author using a predetermined answer key (Appendix H). All 12 questions were weighed equally, and no partial credit was provided for partly correct responses. Correct responses were given a score of 1 and incorrect responses a score of 0. The highest possible score was 12 (100%). Interns had an average of 85% correct, with scores ranging from 67% to 100%.

## Discussion

The transition between medical student to intern is difficult and overwhelming without appropriate guidance. Several studies have demonstrated decreased quality of patient care in teaching hospitals at the beginning of the academic year.^3^ A dedicated curriculum to handle routine inpatient nurse pages can provide internal medicine interns with the skills necessary to facilitate this transition and improve patient care.^5,6^ Our mock paging curriculum is unique in that it has been specifically designed to address topics that interns personally reported as difficult and frequently encountered. Compared to lectures that teach knowledge through passive learning, our curriculum improves both knowledge and skills by actively engaging interns through role-playing and direct feedback from peers. The curriculum explicitly focuses on practical and relevant information interns need to manage these acute inpatient issues. It also allows interns to practice effective communication with nurses, which is important as they establish professional relationships with ancillary staff.

Interns enjoyed participating in the curriculum and found it to be a valuable learning experience. They provided positive feedback about how they enjoyed the small-group setting and the interactive nature of the curriculum. Our results demonstrate that the curriculum was effective in teaching them knowledge and skills to manage inpatient scenarios. After participating in our curriculum, interns reported increases in knowledge, skill set, and comfort in managing common issues on medicine wards. On a 2-week follow-up knowledge test, interns were able to apply the knowledge and skills they had learned in the curriculum to new patient scenarios, indicating that they had retained the material discussed. As the curriculum is implemented in future years, we plan to obtain additional data on its effectiveness.

Our intervention focuses on improving medicine interns’ comfort, practical knowledge, and skills in handling acute scenarios involving nurse pages at the beginning of intern year. While there is another curriculum^20^ focusing on improving interns’ management of common internal medicine issues, efficiency, and communication skills, its sessions span more than the first 3 months of the academic year whereas interns may require some of these skills earlier. When providing feedback on our curriculum, several interns felt that these sessions should be offered during orientation if possible. If taught a significant amount of material early on, interns can immediately use the information, which can help decrease the adverse outcomes associated with the transition period at the beginning of the academic year. There are similar curricula^11^ for fourth-year medical students, but students are not yet physicians and not independently using these skills. Interns directly face the responsibilities of becoming a new physician, and thus, these skills are likely more applicable at this time.

While most other active curricula involve high-fidelity mannequin simulation, ours allows for interaction and engagement without any equipment. Additionally, facilitators do not need any specific preparation or training beforehand apart from familiarizing themselves with the curriculum. One limitation of our curriculum is that it requires residents to volunteer as facilitators. In order to preserve the small-group format in larger residency programs, a greater number of resident facilitators would be needed, which may not always be feasible. Interns also need dedicated time away from clinical duties to participate in these sessions. Most residency programs have dedicated didactic time for house staff, but there may be interns rotating at other sites than the home institution or night shifts that may not be able to participate. In addition, although our results demonstrated that the curriculum increased interns’ knowledge and skills in managing inpatient scenarios, this result was based on only one intern class. Additional data will be needed in future years to better determine the curriculum's effectiveness. Some of the questions in our survey were also positively skewed, and this could have influenced interns’ responses, especially at the beginning of the academic year.

Future considerations for other programs adapting this curriculum include spacing the sessions by 1–2 weeks as spaced repetition may be a more effective means of remembering the information. Furthermore, our curriculum focuses on teaching interns skills to handle acute inpatient scenarios. In order to adequately discuss these skills in a limited time frame, the curriculum does not contain in-depth details on each of the paging issues but rather focuses on practical information and skills that interns can readily apply in the first week of their training.

Our interactive curriculum provides medicine interns in the first week of their residency with knowledge and skills to independently manage common ward issues. This subsequently helps interns feel more prepared for their clinical responsibilities and can potentially reduce adverse patient outcomes. As the curriculum involves active learning through role-playing, interns are able to retain the information taught, as demonstrated by our knowledge test results. Given that no additional materials are required, we expect this curriculum to be easily implemented at other institutions.

## Appendices

Intern Guide Day 1.docxIntern Guide Day 2.docxFacilitator Guide Day 1.docxFacilitator Guide Day 2.docxEKG for Tachycardia Case.pdfSession Evaluation.docxKnowledge Test.docxAnswer Key for Knowledge Test.docx
All appendices are peer reviewed as integral parts of the Original Publication.
