# Supplementary material for: An Interactive Mock Paging Curriculum to Prepare New Internal Medicine Interns for Inpatient Wards
Source: MedEdPORTAL. 2021 Jan 13;17:11082. doi: 10.15766/mep_2374-8265.11082 (PMC7809929; doi:10.15766/mep_2374-8265.11082)
Supplement: Supplementary file 1 — Intern Guide Day 1.docxIntern Guide Day 2.docxFacilitator Guide Day 1.docxFacilitator Guide Day 2.docxEKG for Tachycardia Case.pdfSession Evaluation.docxKnowledge Test.docxAnswer Key for Knowledge Test.docx [file mep_2374-8265.11082-s001.zip › A. Intern Guide Day 1.docx]

**Intern Boot Camp: Mock Paging Session #1**

**Intern Guide**

**Session Goals**

- Improve ability to manage common issues encountered on inpatient medicine wards
- Identify if an issue is urgent or emergent and recognize which patients require immediate attention

**Session Structure**

- Interns will take turns playing the role of a cross covering intern and answer pages based on sign out provided
- Other interns will observe and provide feedback
- Facilitator will play the role of the floor RN providing the page
- Following each mock page, there will be a brief discussion including relevant teaching points and feedback

**Role as Intern**

- When addressing a page, determine urgency of the issue; patients that are unstable should be seen urgently
- Practice communicating effectively with nurses and maintain a professional attitude
  - Introduce yourself: “Hi my name is X, I am the intern taking care of Patient Z, how can I help?”
  - Respond politely and in a timely manner
  - Demonstrate willingness to help
- Gather pertinent information from the nurse which can include vital signs, current clinical status and other significant symptoms
- Clearly communicate your plan to the nurse and provide specific orders
  - Providing both your assessment and plan will help the nurse understand your perspective (“I think his shortness of breath may be related to his COPD exacerbation but the acute change is concerning. I will order a chest x-ray and meet you at the bedside.”)
  - For urgent situations, can provide verbal orders and let them know that you will come see the patient (“Please start 1L of normal saline now and I will come see the patient”)
  - If relevant, communicate when the nurse should contact you again (“I will order acetaminophen for this patient now and please let me know if his pain is not controlled in 2 hours”)
- For patients that are unstable, consider discussing the case with the senior resident
- For each case, discuss what documentation (if any) is necessary
  - In general, you should write an event note for patients with a change in clinical status, change in management plan (with placement of > 1 order) or patients that you see.

**Session 1** (1 hour)

1. Brief overview on session structure (2 minutes)
2. Mock pages (56 minutes)
3. Wrap up: final questions and feedback for group (2 minutes)

**Sign Out**

**BV** is a 61-year-old man with HTN, HLD, and PAD who presented with chest pain, admitted for NSTEMI. EKG showed some non-specific ST changes, troponin peaked at 7. On heparin drip, waiting for left heart catheterization tomorrow.

**CB** is a 58-year-old man with COPD, CAD, and alcoholic cirrhosis who was admitted for hepatic encephalopathy in setting of medication discontinuation. H/H yesterday stable but today down trended from 13 to 11 so follow up repeat H/H tonight. No evidence of GIB currently.

**CO** is a 63-year-old woman with history of COPD on home 2L NC, CAD s/p DES in 2009, HTN, and HLD who presented with shortness of breath and cough, found to have new RLL pneumonia and treating for COPD exacerbation. Currently on 3L NC, ceftriaxone/azithromycin and prednisone.

**DM** is a 57-year-old man with COPD on home 2L NC, CAD and diabetes here with shortness of breath and worsening cough likely due to community acquired pneumonia and COPD exacerbation. Stable on home O2 2L NC, ceftriaxone/azithromycin, methylprednisolone and nebs.

**HN** is a 76-year-old woman with history of mild dementia, CKD, afib, and HTN who presented after a fall, found to have right femoral neck fracture, s/p ORIF without complications and now waiting for rehab bed.

**JP** is a 64-year-old woman with history of hypertension, OA and DM here w/ left knee prosthetic joint infection. Stable on cefazolin, plan for knee wash out with ortho tomorrow.

**OT** is a 67-year-old Cantonese speaking man with HTN and GERD who presented with fever and cough, found to have LLL pneumonia, started ceftriaxone/azithromycin and standing nebs.

**PC** is an 82-year-old woman with dementia, diabetes mellitus, and CKD who presented from nursing facility with confusion and found to be febrile with leukocytosis. Most likely source UTI. Received 1 liter IVF in ED and started on ceftriaxone.

**PD** is 78-year-old woman with history of hypothyroidism and diabetes mellitus admitted this evening for abdominal pain and fevers, found to have cholecystitis. Currently on antibiotics, intravenous fluids and NPO.

**RM** is a 57 year-old-man with ischemic cardiomyopathy (EF 10%), HTN, CAD s/p CABG, afib on rivaroxaban and CKD who presented with dyspnea on exertion and lower extremity swelling, have been treating for heart failure exacerbation with furosemide boluses.

**SN** is a 59-year-old man with history of hypertension and recent diagnosis of AML admitted for induction chemotherapy. Tolerating chemotherapy well so far, not neutropenic.

**TF** is a 47-year-old woman with history of hypothyroidism who presented with abdominal pain and found to have pyelonephritis. On ciprofloxacin, urine culture pending.
