# Supplementary material for: An Interactive Mock Paging Curriculum to Prepare New Internal Medicine Interns for Inpatient Wards
Source: MedEdPORTAL. 2021 Jan 13;17:11082. doi: 10.15766/mep_2374-8265.11082 (PMC7809929; doi:10.15766/mep_2374-8265.11082)
Supplement: Supplementary file 1 — Intern Guide Day 1.docxIntern Guide Day 2.docxFacilitator Guide Day 1.docxFacilitator Guide Day 2.docxEKG for Tachycardia Case.pdfSession Evaluation.docxKnowledge Test.docxAnswer Key for Knowledge Test.docx [file mep_2374-8265.11082-s001.zip › C. Facilitator Guide Day 1.docx]

**Intern Boot Camp: Mock Paging Session # 1**

**Facilitator Guide**

**Session Goals**

- Improve ability to manage common pages on inpatient medicine wards
- Identify if an issue is urgent or emergent and recognize which patients require immediate attention

**Session Structure**

- Interns will take turns playing the role of a cross covering intern and answer pages based on sign out provided
- Other interns will observe and provide feedback
- Facilitator will play the role of the floor RN providing the page
- Following each mock page, there will be a brief discussion including relevant teaching points and possible feedback to the intern

**Role as Facilitator**

- Only provide information asked by the intern
- Encourage interns to practice effective communication techniques with nurses by introducing themselves, maintaining a professional attitude, and providing a clear and specific plan for the patient including whether or not they will come see the patient
- Provide scaffolding by prompting intern or providing clues when not following key tasks
- If asked questions that you are not provided the answers for, feel free to make up normal values or respond that you do not know
- During discussion/debriefing
  - Ask other interns what they thought the role playing intern had done well
  - Provide tips on what the intern could improve on next time including their communication
  - Review remainder of teaching points that were not yet discussed
  - Discuss what type of documentation (if any) is needed in each specific case

**Session 1** (1 hour)

1. Brief overview on session structure (2 minutes)
2. Fever case (7 minutes)
3. Hypotension in setting of bleeding case (10 minutes)
4. Tachycardia case (10 minutes)
5. Hypertension case (7 minutes)
6. Hypotension in setting of sepsis case (12 minutes)
7. Sleep case (5 minutes)
8. Wrap up: answer final questions and feedback for group (2 minutes)
9. **Fever case** (7 minutes)

**SIGN OUT**: TF is a 47-year-old woman with history of hypothyroidism who presented with abdominal pain and found to have pyelonephritis. On ciprofloxacin, urine culture pending.

**PAGE**: TF is febrile to 38.7, not ordered for any antipyretics.

***Notes for nurse:***

- - If asked for full set of vitals: T 38.7 BP 117/82 HR 123 RR 20 O2 sat 97% room air
  - Previously patient afebrile, BP 120s/80s HR 80
  - Patient feels warm, slightly uncomfortable due to fever but no other new symptoms
  - Last blood cultures were drawn yesterday on day of admission and remain negative

***Desired learner actions:***

- - Ask for full set of current vital signs
  - Inquire about patient’s symptoms and clinical status
  - Inform nurse that you will order acetaminophen and repeat cultures including blood culture, urine culture; consider chest x-ray if respiratory symptoms
  - Could consider seeing patient
  - Documentation: consider writing brief event note with the patient’s vitals, symptoms and orders placed

***Teaching points:***

- - For any patient with significant fever overnight, make sure repeat blood cultures (if > 24 hours) and urine culture drawn. Chest x-ray and sputum culture as well if respiratory symptoms or source unknown.
  - Obtain full set of vital signs to make sure no concern for sepsis.
  - Examine patient to determine source of fever or make sure exam stable if source already known
  - *When do you start antibiotics?* If patient clinically unstable, start antibiotics preferably directed towards source or if already on antibiotics, broaden to cover additional organisms. If patient otherwise clinically stable on current antibiotic regimen and low grade fever, do not necessarily need to broaden antibiotics.

1. **Hypotension in setting of bleeding** (10 minutes)

**SIGN OUT**: Patient CB is a 58-year-old man with COPD, CAD, and alcoholic cirrhosis who was admitted for hepatic encephalopathy in setting of medication discontinuation. H/H yesterday stable but today down trended from 13 to 11 so follow up repeat H/H tonight. No evidence of GIB currently.

**PAGE**: Please call re: Pt CB repeat Hb 9.4, BP 86/60.

*You check the rest of his lab work which shows WBC 10 (stable), platelet count 90 (stable), and BUN 30 (stable).*

***Notes for nurse:***

- - Remainder of patient’s vitals: T 36.7 BP 86/60 HR 110s RR 20 O2 sat 90% on room air; previously BP 100s/70s
  - Patient has had two large black bowel movements in last few hours
  - Feels lightheaded but no abdominal pain
  - No known history of esophageal varices
  - IV access: one 22g PIV, one 20g PIV

***Desired learner actions:***

- - Obtain full set of current vital signs
  - Assess if patient having any symptoms or signs of active bleeding
  - Determine IV access patient currently has
  - Ask to obtain more IV access and other lab work including type and screen, INR, repeat H/H
  - Transfuse blood and give IVF; start IV pantoprazole for possible upper GI bleed
  - Ask about history of esophageal varices, consider starting octreotide
  - Communicate to the nurse that you are concerned about this patient and you will come assess them urgently
  - Discuss patient with senior resident, consider calling GI fellow after evaluating patient
  - Documentation: as this patient now has hemodynamic instability, important to write an event note describing patient’s melena, vital signs, orders placed and assessment/plan

***Teaching points:***

- - In any patient with acute drop in hemoglobin, need to first obtain full set of vital signs and determine if symptomatic
  - If suspect significant GI bleed, ensure patient has appropriate access: at least two large bore (18g) IVs in place. Try to evaluate the bleeding yourself (e.g. look at melena to determine amount/significance)
  - Patient should receive IVF or blood if actively bleeding; ensure active type and screen
  - For upper GI bleed: start IV PPI (pantoprazole 40mg IV BID versus drip); if variceal bleed: octreotide bolus followed by infusion and ceftriaxone (for SBP prophylaxis)
  - Maintain NPO and discuss case with GI fellow immediately

1. **Tachycardia Case** (10 minutes)

**SIGN OUT**: OT is a 67-year-old Cantonese speaking man with HTN and GERD who presented with fever and cough, found to have LLL pneumonia, started ceftriaxone/azithromycin and standing nebs.

**PAGE**: Patient OT HRs up to 130s.

***Notes for Nurse:***

- - If asked for full set of vitals: T 38.3 BP 132/85 HR 135 RR 20 O2 sat 98% 2L NC
  - Admission EKG showed sinus tachycardia in 90s but in last 10-15 minutes up to 130s.
  - Patient feels like heart racing but no chest pain or shortness of breath currently.
  - Last nebulizer treatment was about an hour ago

***Desired Learner Actions:***

- - Obtain full set of vital signs
  - Obtain more information about tachycardia – previous heart rates and EKG
  - Inquire about current clinical status
  - Order EKG and telemetry monitoring
  - Inquire about recent medications; stop standing albuterol and consider ipratropium nebulizers alone if needed
  - Notify nurse you will come see patient. Ask for an in-person interpreter (if available) or remote interpreter via telephone.

**3B. EKG Interpretation (switch to another intern)**

When you examine patient, they appear mildly uncomfortable. Exam notable for tachycardia, no murmurs and decreased breath sounds over left base. ***Share EKG (Appendix H)****.*

***Desired learner actions:***

- - Interpret EKG as atrial fibrillation with RVR – note irregularly irregular rhythm, absent P waves
  - Order metoprolol 5mg IV, monitor blood pressure following this
  - Start metoprolol PO 30 minutes later
  - Order chemistry and replete potassium and magnesium
  - Documentation: this patient has new atrial fibrillation and is being started on new medications. Need to write event note describing events, patient’s symptoms, and interventions performed

***Teaching Points:***

- - New onset atrial fibrillation can occur during illness (sepsis, fevers) or exacerbated by medications.
  - If patient is hemodynamically unstable (hypotensive), next step is cardioversion.
  - If patient is hemodynamically stable (BP stable), trial pharmacologic treatment with rate control agents such as beta blockers or calcium channel blockers. Start with intravenous forms which are short acting to immediately decrease heart rates and then start oral form for maintenance (peak effect of metoprolol IV occurs in 20 minutes; peak effect of oral metoprolol in 1-2 hours). If first time new onset atrial fibrillation, consider discussing early cardioversion with cardiology.
  - Avoid calcium channel blockers in patients with known HFrEF; avoid beta blockers in cardiogenic shock
  - Anticoagulation should be discussed based on CHADs2-VASC score (if episodes occurs during night float, can defer decision for anticoagulation to day)
  - Typically, replete electrolytes to K > 4, Mg > 2
  - It is important to verify history obtained from nurse, especially in patients with limited English proficiency as it may change management. If during daytime hours, try to arrange for an in-person interpreter but if acute situation, could use remote interpreter via telephone.

1. **Hypertension Case** (7 minutes)

**SIGN OUT**: HN is a 76-year-old woman with history of mild dementia, CKD, afib, and HTN who presented after a fall, found to have right femoral neck fracture, s/p ORIF without complications and now waiting for rehab bed.

**PAGE**: Patient HN, BP is 165/98, any intervention?

***Notes for nurse:***

- - Full set of vital signs: T 36.7 BP 165/98 HR 96 RR 16 O2 sat 100% RA
  - If asked for recheck: 168/105
  - Previous BPs: during day 150s/90s, day team had already increased her morning lisinopril dose; BPs uptrended over evening to now 160s/100s
  - Patient does not have any pain currently, appears comfortable; no headaches, vision changes or chest pain

***Desired learner actions:***

- - Obtain full set of current vital signs and repeat blood pressure
  - Inquire about BP trend
  - Determine if patient in pain or symptomatic from high blood pressure
  - Communicate to nurse that as the blood pressure elevation is chronic and patient is asymptomatic, does not need to currently be treated
  - Consider increasing morning lisinopril dose or if at max dose, advising team to add second anti-hypertensive
  - Documentation: could consider writing brief event note but not necessary

***Teaching points:***

- - When evaluating elevated blood pressures, determine if acute or chronic elevation, and if could be due to other causes (pain, alcohol withdrawal, opioid withdrawal, constipation, urinary retention, missing antibiotic doses among others).
  - Patients with chronically elevated blood pressures do not need rapid correction especially if asymptomatic or elderly
  - For patients who require rapid correction of blood pressures (patient with chest pain and NSTEMI, hypertensive emergency, or recent stroke) or unable to tolerate oral intake, consider topical nitroglycerin, IV hydralazine or labetalol. Increase long acting oral medications to maintain consistent blood pressure control as well.

1. **Hypotension in setting of sepsis** (12 minutes)

**SIGN OUT**: PC is an 82-year-old woman with dementia, diabetes mellitus, and CKD who presented from nursing facility with confusion and found to be febrile with leukocytosis. Most likely source UTI. Received 1 liter IVF in ED and started on ceftriaxone.

**PAGE**: Please call re: Pt PC, BP 85/48, repeat 82/50.

***Notes for nurse:***

- - Remainder of current vitals: T 38.4 BP 85/48 (repeated twice with correct cuff) HR 118 O2 sat 95% room air RR 20
  - If asked about trend in vitals – blood pressure previously in ED 90s/60s and after 1 liter IVF 100s/60s; HR has been 100s-110s, sinus
  - Patient confused, only oriented to person but responding to some questions, following commands
  - Already received ceftriaxone in ED prior to transfer to floor
  - IV access: 20 g PIV

***Desired learner actions***

- - Obtain full set of vital signs
  - Ask about patient’s current clinical status and if significant changes from prior
  - Determine IV access patient currently has
  - Administer more IVF (roughly 30cc/kg) and acetaminophen for fever; if not already recently obtained, make sure patient has blood cultures pending
  - Obtain repeat CBC, lactate, renal function
  - Consider broadening antibiotics
  - Notify nurse that you are concerned that this patient is septic and will come see patient
  - Discuss patient with senior resident
  - Documentation: as this patient has hemodynamic instability, necessary to write event note describing patient’s trend in blood pressure, brief assessment (e.g. hypotension likely due to sepsis) and orders placed

***Teaching points***

- - If suspect hypotension related to sepsis, first step is fluid resuscitation. *How much?*
    1. Roughly 30cc/kg preferably in first three hours
  - *What clinical signs and lab work can help determine degree of shock?*
    1. Mental status, lactate, renal function, urine output
  - Need to monitor vitals and lab work closely with fluid resuscitation: repeat vitals every 30 minutes to an hour, lactate every 4 hours until normalization. If history of cardiac disease, heart failure, or end stage renal disease, also need to monitor for volume overload.
  - If patient adequately fluid resuscitated and still hypotensive, next step would be to initiate pressors. First line in septic shock is norepinephrine.
  - If source is unknown or suspect that other pathogens may not be covered (ESBL in this case), consider broadening antibiotics.
  - Another key aspect of septic shock is source control. In this case, source is assumed to be urinary but if unknown, consider further work up to evaluate. In immediate situation, chest x-ray and symptoms can be helpful. Once patient more stable, imaging could also be considered (ex: CT abdomen/pelvis to look for potential abscess that may need drainage)

1. **Sleep case** (5 minutes)

**SIGN OUT**: Patient SN is a 59-year-old man with history of hypertension and recent diagnosis of AML admitted for induction chemotherapy. Tolerating chemotherapy well so far, not neutropenic.

**PAGE**: Patient SN unable to sleep, already received melatonin. Can we try something else?

***Notes for Nurse:***

- - Patient otherwise stable, no new complaints
  - He has been having issues sleeping last few nights
  - Only ordered for melatonin PRN; no benzodiazepines or other medications for insomnia

***Desired Learner Actions:***

- - Inquire about overall clinical status and mental status
  - Notify nurse you will order trazodone
  - Documentation: not necessary to write note given low acuity of symptoms and lack of major intervention

***Teaching Points:***

- - Sleep disturbances are a common issue in hospitalized patients
  - Important to differentiate between issues with sleeping versus anxiety (often related to upcoming procedure)
  - Melatonin could be used at first but may not see clear response
  - Trazodone is generally safe in elderly population
  - Avoid benzodiazepines and diphenhydramine in elderly due to side effects especially delirium
  - Zolpidem should be avoided in patients with liver disease or elderly
