# Supplementary material for: An Interactive Mock Paging Curriculum to Prepare New Internal Medicine Interns for Inpatient Wards
Source: MedEdPORTAL. 2021 Jan 13;17:11082. doi: 10.15766/mep_2374-8265.11082 (PMC7809929; doi:10.15766/mep_2374-8265.11082)
Supplement: Supplementary file 1 — Intern Guide Day 1.docxIntern Guide Day 2.docxFacilitator Guide Day 1.docxFacilitator Guide Day 2.docxEKG for Tachycardia Case.pdfSession Evaluation.docxKnowledge Test.docxAnswer Key for Knowledge Test.docx [file mep_2374-8265.11082-s001.zip › D. Facilitator Guide Day 2.docx]

**Intern Boot Camp: Mock Paging Session # 2**

**Facilitator Guide**

**Session Goals**

- Improve ability to manage common pages on inpatient medicine wards
- Identify if an issue is urgent or emergent and recognize which patients require immediate attention

**Session Structure**

- Interns will take turns playing the role of a cross covering intern and answer pages based on sign out provided
- Other interns will observe and provide feedback
- Facilitator will play the role of the floor RN providing the page
- Following each mock page, there will be a brief discussion including relevant teaching points and possible feedback to the intern

**Role as Facilitator**

- Only provide information asked by the intern
- Encourage interns to practice effective communication techniques with nurses by introducing themselves, maintaining a professional attitude, and providing a clear and specific plan for the patient including whether or not they will come see the patient
- Provide scaffolding by prompting intern or providing clues when not following key tasks
- If asked questions that you are not provided the answers for, feel free to make up normal values or respond that you do not know
- During discussion/debriefing
  - Ask other interns what they thought the role playing intern had done well
  - Provide tips on what the intern could improve on next time including their communication
  - Review remainder of teaching points that were not yet discussed
  - Discuss what type of documentation (if any) is needed in each specific case

**Session 2** (1 hour)

1. Brief overview on session structure (2 minutes)
2. Pain case (7 minutes)
3. Shortness of breath due to COPD case (12 minutes)
4. Chest pain case (15 minutes)
5. Hyperglycemia case (7 minutes)
6. Shortness of breath due to heart failure case (7 minutes)
7. Hyperglycemia in setting of NPO case (5 minutes)
8. Wrap up: answer final questions and feedback for group (2 minutes)
9. Survey (5 minutes)
10. **Pain case** (7 minutes)

**SIGN OUT**: Patient PD is 78-year-old woman with history of hypothyroidism and diabetes mellitus admitted this evening for abdominal pain and fevers, found to have cholecystitis. Currently on antibiotics, intravenous fluids and NPO.

**PAGE**: PD 8/10 abdominal pain, already received acetaminophen. Can she get something else for pain?

***Notes for nurse:***

- Patient has been clinically stable
- Current vitals: T 37.1 BP 147/88 HR 98 RR 20 O2 sat 95% RA
- Pain similar in intensity to prior, no worsening
- No other new symptoms
- Only ordered for acetaminophen, no other medications

***Desired learner actions:***

- Assess patient’s vital signs
- Inquire about mental status and overall clinical status
- Consider examining patient prior to giving pain medication if appears to be clinically worsening
- Once established that patient clinically stable, given elder age, trial lower dose opioids (tramadol or oxycodone) first.
- Clearly communicate to nurse your plan to reassess patient’s pain later on
- Documentation: consider writing brief event note with the patient’s vitals, symptoms and orders placed

***Teaching points:***

- Prior to giving pain relievers for abdominal pain, make sure abdominal exam and patient clinically stable. Could consider repeat KUB to rule out perforation.
- Vital signs can be useful in judging severity of pain though be mindful that patients on nodal blockers may not be tachycardic. Pupil size can also be helpful as dilated pupils can indicate increased sympathetic tone in setting of pain. Other causes of pain such as urinary retention or constipation should also be considered.
- In elderly patients, if pain medication needed, trial lower dose opiates (tramadol, oxycodone, hydromorphone) first. Preferably oral if able to tolerate.
- If AKI or CKD, avoid oxycodone and morphine since metabolites can accumulate and contribute to altered mental status especially in the elderly. Tramadol can lower seizure threshold.
- After pain medication administered, re-examine patient to see if responded. If still with significant pain, consider alternative etiologies or medications.

1. **Shortness of breath due to COPD case** (12 minutes)

**SIGN OUT**: CO is a 63-year-old woman with history of COPD on home 2L NC, CAD s/p DES in 2009, HTN, and HLD who presented with shortness of breath and cough, found to have new RLL pneumonia and treating for COPD exacerbation. Currently on 3L NC, ceftriaxone/azithromycin and prednisone.

**PAGE**: CO more SOB, now up to 5L NC.

***Notes for nurse:***

- If asked for full set of vitals: T 37.4 BP 134/86 HR 105 RR 28 O2 sat 95% on 5L NC
- Previous vitals similar except for RR 18 and on 3L NC
- Patient appears to be working harder to breathe, uncomfortable; some wheezing on exam
- Has albuterol/ipratropium nebulizer ordered but have not tried this yet

***Desired learner actions:***

- Obtain full set of current vital signs
- Inquire about respiratory status
- Inform nurse that you are concerned about patient. They can administer albuterol/ipratropium nebulizer and you will come see patient urgently
- Order chest x-ray and ABG

**2B. Further prompting (can switch to another intern)**

When you examine the patient, they appear tachypneic with increased work of breathing and accessory muscle use. They are responding to some questions but appear very fatigued. Exam notable for diffuse wheezing, decreased air movement and crackles at right base. Chest x-ray similar to prior with hyperinflated lungs and RLL infiltrate. ABG with pH: 7.25, pCO2: 65 (assume remainder of ABG is normal). What would you do next? How would you support their respiratory status?

***Desired learner actions:***

- Interpret ABG as respiratory acidosis
- Consider calling respiratory therapy to set up bipap
- Order standing albuterol/ipratropium nebulizers
- After stabilizing patient, also consider further evaluation for pulmonary embolism
- Discuss patient with senior resident
- Documentation: write event note describing patient’s symptoms, vital signs/exam, and assessment/plan including orders placed

***Teaching points:***

- For all patients with changes in respiratory status, evaluation includes full set of vitals, physical examination, chest x-ray, and arterial blood gas.
- If primary issue with hypoxemia (e.g. pneumonia), increase oxygen delivery via nasal cannula, face mask (if mouth breathing); can escalate to high flow nasal cannula if needed.
- If primary issue with hypercapnia (e.g. COPD exacerbation, pulmonary edema with hypercapnia, increased work of breathing), start bipap. *Typical initial settings*: IPAP (inspiratory pressure)/EPAP (expiratory pressure)= 12/8 (can also set RR, FiO2)
- For patients that are unable to protect their airway or concern that there is not enough time to trial non-invasive ventilation, urgent intubation may be needed.
- Treatment of COPD exacerbation involves steroids, duonebs and possible antibiotics. If patient unable to take oral medications, can change prednisone to methylprednisolone.

1. **Chest pain case** (15 minutes)

**SIGN OUT**: BV is a 61-year-old man with HTN, HLD, and PAD who presented with chest pain, admitted for NSTEMI. EKG showed some non-specific ST changes, troponin peaked at 7. On heparin drip, waiting for left heart catheterization tomorrow.

**PAGE**: Please call re: BV, 10/10 chest pain.

***Notes for nurse:***

- Patient was previously sleeping comfortably but then developed sudden onset chest pain that woke him from sleep
- Chest pain is 10/10 substernal pressure and patient appears uncomfortable, diaphoretic, nauseous
- Current vitals: T 36.9 BP 153/98 HR 100s RR 22 O2 sat 96% room air
- Has sublingual nitroglycerin ordered but has not received anything for chest pain yet

***Desired learner actions:***

- Obtain current set of vital signs
- Inquire about nature of chest pain
- Ask nurse to obtain EKG now and administer sublingual nitroglycerin
- Notify nurse you are worried about acute coronary syndrome and will come see patient urgently
- Could also order repeat troponin and chest x-ray (either before or after seeing patient)
- Consider discussing patient with senior resident and seeing patient together
- Documentation: write event note describing patient’s chest pain, vital signs, and assessment/plan including orders placed

***Teaching points:***

- Patients with chest pain that seems to be cardiac in nature should be seen urgently. Vital signs, history, exam, EKG and chest x-ray are all an important part of evaluating chest pain.
- Many pages about chest pain may be non-urgent (heartburn, musculoskeletal) but important to determine nature of chest pain and overall clinical picture (vital signs) to determine urgency and if cardiac.
- After evaluating patient, compare current EKG to prior. If new ST elevations, call cardiology fellow immediately to discuss intervention.
- If EKG shows other ST changes or no changes, but concerned for ACS, initiate therapy immediately. *What does this include?*
  - Full dose aspirin 325mg
  - Heparin bolus + full dose drip (if already on heparin drip, make sure PTT therapeutic)
  - High intensity statin
  - Beta blocker (if no signs of cardiogenic shock)
  - Oxygen to maintain O2 saturation > 90%
  - Monitor troponins (every 8 hours till peak)
- Control patient’s chest pain, goal is for patient to be chest pain free. *What are possible interventions for this?*
  - Start with sublingual nitroglycerin (can give three tablets a few minutes apart)
  - If still with chest pain, consider topical nitroglycerin ointment, morphine; if unable to control chest pain with these therapies, next step would be escalating to nitroglycerin drip
  - Monitor blood pressure with above therapies
  - If still having significant chest pain despite these interventions, discuss with cardiology fellow as may need more emergent catheterization

1. **Hyperglycemia case** (7 minutes)

**SIGN OUT:** Patient DM is a 57-year-old man with COPD on home 2L NC, CAD and diabetes here with shortness of breath and worsening cough likely due to community acquired pneumonia and COPD exacerbation. Stable on home O2 2L NC, ceftriaxone/azithromycin, methylprednisolone and nebs.

**PAGE**: Pt DM bedtime BG 252, not ordered for anything, any additional insulin?

***Notes for nurse:***

- Only ordered for insulin sliding scale with meals. At home on high dose metformin which has been held inpatient
- Trend of blood sugar today: 200s-250s, requiring 4-5 units insulin
- Ordered for regular diet

***Desired learner actions:***

- Determine current insulin regimen and identify how much insulin patient has been requiring
- Order insulin for current finger stick
- Increase sliding scale to high dose and switch patient to basal/bolus regimen
- Inquire about diet, change to diabetic
- Documentation: not necessary to write an event note but communicate patient’s hyperglycemia to primary team at sign out

***Teaching points:***

- Steroids can result in elevated blood sugars
- Insulin requirement should be monitored daily and adjusted based on finger sticks
- Exclusive use of sliding scale discouraged; preferred insulin regimen is basal/bolus especially if expect long hospitalization or longer duration of steroids
- How do you calculate a patient’s insulin requirement? Total daily insulin requirement is roughly 0.5 units/kg/day. This can be divided into basal (50%) and bolus/prandial (50% which is then divided by 3 for each meal)
- Goal finger sticks: < 140 premeal, < 180 random

1. **Shortness of breath due to heart failure case** (7 minutes)

**SIGN OUT**: RM is a 57 year-old-man with ischemic cardiomyopathy (EF 10%), HTN, CAD s/p CABG, afib on rivaroxaban and CKD who presented with dyspnea on exertion and lower extremity swelling, have been treating for heart failure exacerbation with furosemide boluses.

**PAGE**: RM feeling more SOB, putting on 2L NC

***Notes for nurse:***

- If asked for full set of vitals: T 37.2 BP 142/84 HR 96 RR 20 O2 sat 88% RA, 97% on 2L NC
- Previous vitals similar except 95% on RA
- If asked for I/Os today: 2300/1100= +1.2L
- If asked about respiratory status, SOB has been gradually increasing over the evening; appears to be uncomfortable but not using accessory muscles
- Patient has been receiving furosemide 80mg IV boluses

***Desired learner actions:***

- Obtain full set of current vital signs
- Inquire about respiratory status and I/Os for last 24 hours
- Order chest x-ray, EKG; consider ABG if tachypneic, increased work of breathing
- Inform nurse that you will come see patient urgently and that their symptoms are most likely due to volume overload
- Discuss potential diuretics you will administer (furosemide 120mg IV bolus and/or drip)
- Consider bipap
- Documentation: important to write an event note describing patient’s change in respiratory status, vitals, labs/imaging performed and assessment/plan including specific orders

***Teaching points:***

- For shortness of breath from heart failure exacerbation, evaluation involves physical exam and chest x-ray; EKG, ABG, troponin could also be considered
- Important to control blood pressures as hypertension can lead to flash pulmonary edema
- Start with furosemide boluses (IV is twice as concentrated as PO). Monitor urine output closely. If urine output does not increase in 1-2 hours, consider escalating diuretic to either furosemide drip or adding chlorothiazide.
- If tachypneic and increased work of breathing, can consider bipap for respiratory support until diuretics take effect. If no improvement with diuretics and bipap, also consider pulmonary embolism given acute change in clinical status.
- Monitor electrolytes and renal function with diuresis

1. **Hyperglycemia in setting of NPO case** (5 minutes)

**SIGN OUT**: Patient JP is a 64-year-old woman with history of hypertension, OA and DM here w/ left knee prosthetic joint infection. Stable on cefazolin, plan for knee wash out with ortho tomorrow.

**PAGE**: JP due for 30 units insulin glargine tonight but NPO at midnight, ok to give?

***Notes for nurse:***

- Patient ordered for 30 units insulin glargine at night and high dose lispro insulin sliding scale with meals
- Current FS 164; range during hospitalization has been 120 to 190 (while receiving glargine) and morning FS 120-140

***Desired learner actions:***

- Inquire about patient’s current insulin regimen
- Determine trend of patient’s finger sticks during hospitalization; make sure no recorded hypoglycemic events
- Clearly communicate to nurse that they should still give basal insulin (either at same dose or reduced dose) and switch insulin sliding scale from lispro to regular; hold any mealtime insulin
- Documentation: do not need to write an event note for this intervention but communicate changes to primary team at sign out

***Teaching points:***

- When a patient is NPO, mealtime insulin (bolus) should be held but basal insulin should be continued. If expect patient will be NPO for extended period or worried about hypoglycemia (high insulin dose, borderline hypoglycemic events), can decrease basal insulin dose by 50%
- Longer acting insulin (regular) preferred over short acting (lispro) while NPO
- Should still continue to monitor finger sticks and receive appropriate amount of regular insulin even while NPO
