# Supplementary material for: An Interactive Mock Paging Curriculum to Prepare New Internal Medicine Interns for Inpatient Wards
Source: MedEdPORTAL. 2021 Jan 13;17:11082. doi: 10.15766/mep_2374-8265.11082 (PMC7809929; doi:10.15766/mep_2374-8265.11082)
Supplement: Supplementary file 1 — Intern Guide Day 1.docxIntern Guide Day 2.docxFacilitator Guide Day 1.docxFacilitator Guide Day 2.docxEKG for Tachycardia Case.pdfSession Evaluation.docxKnowledge Test.docxAnswer Key for Knowledge Test.docx [file mep_2374-8265.11082-s001.zip › F. Session Evaluation.docx]

**Intern Boot Camp Mock Paging Sessions: Intern Survey**

1. Overall, how useful was the content of the mock paging sessions?

|  |  |  |  |  |
| --- | --- | --- | --- | --- |
| Not at all useful | Slightly useful | Moderately useful | Quite useful | Extremely useful |

1. How much did these sessions improve your knowledge on managing routine encounters on inpatient wards?

|  |  |  |  |  |
| --- | --- | --- | --- | --- |
| Almost nothing | A little bit | Some | Quite a bit | A tremendous amount |
|  |  |  |  |  |

1. How much did these sessions improve your skills (responding in a timely manner, assessing urgency of issues, etc.) on managing routine encounters on inpatient wards?

|  |  |  |  |  |
| --- | --- | --- | --- | --- |
| Almost nothing | A little bit | Some | Quite a bit | A tremendous amount |
|  |  |  |  |  |

1. How difficult did you find the mock paging sessions?

|  |  |  |  |  |
| --- | --- | --- | --- | --- |
| Not at all difficult | Slightly difficult | Appropriately difficult | Quite difficult | Very difficult |

1. How likely are you to remember the information from these sessions?

|  |  |  |  |  |
| --- | --- | --- | --- | --- |
| Not at all likely | Slightly likely | Somewhat likely | Quite likely | Very likely |

1. How often will you use the information you learned during these sessions on future inpatient ward rotations?

|  |  |  |  |  |
| --- | --- | --- | --- | --- |
| Almost never | Once in a while | Sometimes | Often | Almost always |

1. Please rate how comfortable you felt managing these issues in the first week of intern year ***BEFORE*** these mock paging sessions.

|  | Very uncomfortable | Slightly uncomfortable | Neutral | Pretty comfortable | Very comfortable |
| --- | --- | --- | --- | --- | --- |
| **High blood sugar** | 1 | 2 | 3 | 4 | 5 |
| **Chest pain** | 1 | 2 | 3 | 4 | 5 |
| **Fever** | 1 | 2 | 3 | 4 | 5 |
| **Hypotension** | 1 | 2 | 3 | 4 | 5 |
| **Pain** | 1 | 2 | 3 | 4 | 5 |
| **Tachycardia** | 1 | 2 | 3 | 4 | 5 |
| **Shortness of breath** | 1 | 2 | 3 | 4 | 5 |
| **Hypertension** | 1 | 2 | 3 | 4 | 5 |
| **Difficulty sleeping** | 1 | 2 | 3 | 4 | 5 |

1. Please rate how comfortable you feel managing these issues now ***AFTER*** completing these mock paging sessions.

|  | Very uncomfortable | Slightly uncomfortable | Neutral | Pretty comfortable | Very comfortable |
| --- | --- | --- | --- | --- | --- |
| **High blood sugar** | 1 | 2 | 3 | 4 | 5 |
| **Chest pain** | 1 | 2 | 3 | 4 | 5 |
| **Fever** | 1 | 2 | 3 | 4 | 5 |
| **Hypotension** | 1 | 2 | 3 | 4 | 5 |
| **Pain** | 1 | 2 | 3 | 4 | 5 |
| **Tachycardia** | 1 | 2 | 3 | 4 | 5 |
| **Shortness of breath** | 1 | 2 | 3 | 4 | 5 |
| **Hypertension** | 1 | 2 | 3 | 4 | 5 |
| **Difficulty sleeping** | 1 | 2 | 3 | 4 | 5 |

1. What did you like about these sessions?

____________________________________________________________________________________________________________________________________________________________________________________________________________________________________________________________

____________________________________________________________________________________

1. What would you change about these sessions to better prepare you for intern year?

____________________________________________________________________________________________________________________________________________________________________________________________________________________________________________________________

____________________________________________________________________________________

1. Were there any topics that should be removed from the sessions?

________________________________________________________________________________________________________________________________________________________________________

1. Any topics that would have been helpful to cover?

________________________________________________________________________________________________________________________________________________________________________

1. Overall, I would rate this session as:

|  |  |  |  |  |
| --- | --- | --- | --- | --- |
| Poor | Fair | Good | Very Good | Excellent |

1. Feel free to provide any other feedback for us ☺

________________________________________________________________________________________________________________________________________________________________________________________________________________________________________________________________________________________________________________________________________________
