# Supplementary material for: An Interactive Mock Paging Curriculum to Prepare New Internal Medicine Interns for Inpatient Wards
Source: MedEdPORTAL. 2021 Jan 13;17:11082. doi: 10.15766/mep_2374-8265.11082 (PMC7809929; doi:10.15766/mep_2374-8265.11082)
Supplement: Supplementary file 1 — Intern Guide Day 1.docxIntern Guide Day 2.docxFacilitator Guide Day 1.docxFacilitator Guide Day 2.docxEKG for Tachycardia Case.pdfSession Evaluation.docxKnowledge Test.docxAnswer Key for Knowledge Test.docx [file mep_2374-8265.11082-s001.zip › G. Knowledge Test.docx]

**Intern Boot Camp Mock Paging Sessions: Knowledge Test**

Please answer each of the following questions to the best of your knowledge. This survey will take about 10 minutes to complete.

1. A 59-year-old man with hypertension and diabetes presents with shortness of breath and cough for three days. Lab work shows WBC of 14 and chest x-ray demonstrates hazy opacities on the right lower lobe. He is started on antibiotics for community acquired pneumonia. A few hours later you are paged that the patient is febrile to 39.1. What additional information do you need to ask the nurse?

­­­­___________________________________________________________________________________

­­­­___________________________________________________________________________________

1. You are the night float intern covering the medicine services. A nurse pages you overnight that one of your patients, a 75-year-old man with COPD and BPH, here for COPD exacerbation, is unable to sleep. Melatonin was trialed without effect. Which other medication could you consider for his insomnia?

________________________________________________________________________________________________________________________________________________________________________

1. A 54-year-old woman with alcoholic cirrhosis and COPD presents with abdominal pain. Bedside paracentesis was negative for SBP. Her pain was thought to be due to constipation and she is being treated supportively with plan for discharge tomorrow. This evening, you are paged that her blood pressure is 87/60 and she has had two episodes of melena.
   1. Name at least two diagnostic lab tests you would order.

________________________________________________________________________________________________________________________________________________________________________

- 1. What medications, if any, would you consider starting?

________________________________________________________________________________________________________________________________________________________________________

1. A 72-year-old man with COPD is admitted with fevers, nausea/vomiting and abdominal pain. Lab work and history are consistent with cholangitis. He is started on antibiotics and planned for ERCP tomorrow. Later, his heart rates are in the 130s with stable blood pressure. He feels anxious and describes palpitations. EKG shows atrial fibrillation (patient does not have history of this). What medications would you administer (be specific with medication name, dosage and route)?

________________________________________________________________________________________________________________________________________________________________________

1. A 57-year-old man with hypertension, diabetes, and severe aortic stenosis is admitted with worsening chest pain, likely secondary to his aortic stenosis and planned for TAVR in a few days. You are paged that his pre-lunch blood sugar is 306. Over the last day, his blood sugars have been 200-250s and he has received 8 units insulin. He is usually on home metformin and glipizide which are being held inpatient and he is currently only on medium dose insulin sliding scale. What changes would you consider making to his insulin regimen?

________________________________________________________________________________________________________________________________________________________________________

1. A 62-year-old woman (100kg) with history of GERD and ulcerative colitis is admitted with fevers, and dysuria. CT abdomen/pelvis showed concern for pyelonephritis. She was started on ertapenem and blood cultures were drawn. Her blood pressure in the ED was initially 82/50 but after 1L IVF improved to 90s/60s. Later in the night, the nurse pages you that her blood pressures are 80/60s again with HRs 100s, afebrile.
   1. Would you give her additional fluids or start her on pressors? If additional fluids, how much? If pressor, which one?

________________________________________________________________________________________________________________________________________________________________________

- 1. How much fluid resuscitation is considered adequate for a patient with septic shock?

________________________________________________________________________________________________________________________________________________________________________

1. A 65-year-old woman with history of COPD on home 2L NC presents with worsening dyspnea on exertion and productive cough. Chest x-ray shows left lower lobe infiltrate. She is started on antibiotics for pneumonia and prednisone for COPD exacerbation. A few hours later, you are paged that she is having increased SOB. Vitals are remarkable for RR 28 and O2 sat 95% on 4L NC. On exam, she appears tachypneic with increased work of breathing and some accessory muscle use; diffuse wheezing and decreased air movement at left base.
   1. Name at least two diagnostic tests you would order.

________________________________________________________________________________________________________________________________________________________________________

- 1. What medications, if any, would you consider starting? (Be specific with name, route)

________________________________________________________________________________________________________________________________________________________________________

1. You are admitting a 69-year-old-woman with hypertension and CKD (baseline Cr 2.5) for acute cholangitis. Her vitals on presentation are stable and she is started on antibiotics. The patient has significant abdominal pain that has not been relieved with acetaminophen 650mg two hours ago. What other medications would you consider starting to control this patient’s pain?

________________________________________________________________________________________________________________________________________________________________________

1. A 71-year-old woman with HTN, DM, and HLD presents to the ED with epigastric pain and nausea. Her symptoms were thought to be from GERD, but serial troponins are ordered to rule out MI. Overnight, the patient has recurrent epigastric pain described as 10/10 pressure radiating down both arms and associated with nausea/vomiting. Vital signs significant for BP 157/99 HR 100s. Based on an EKG and troponin, you are concerned for ACS. What medications would you start urgently for this patient?

________________________________________________________________________________________________________________________________________________________________________

____________________________________________________________________________________

____________________________________________________________________________________
